# Association between Self-Reported Bruxism and Sleeping Patterns among Dental Students in Saudi Arabia: A Cross-Sectional Study

**DOI:** 10.1155/2016/4327081

**Published:** 2016-02-29

**Authors:** Shereen M. Shokry, Eman E. El Wakeel, Nassr Al-Maflehi, Zaheera RasRas, Nida Fataftah, Enam Abdul Kareem

**Affiliations:** ^1^Oral Radiology Department, Cairo University, Cairo, Egypt; ^2^Riyadh Colleges of Dentistry and Pharmacy, Riyadh, Saudi Arabia; ^3^Anatomy and Embryology Department, Benha University, Egypt; ^4^Biostatistics Department, King Saud University, Riyadh, Saudi Arabia

## Abstract

*Objectives.* The aim of this cross-sectional study was to identify sleeping patterns among dental students and their association with self-reported bruxism in Riyadh Colleges of Dentistry and Pharmacy (RCsDP).* Methods.* A cross-sectional study was performed including 549 students (67 men and 482 women). A structured questionnaire was adopted from The PSQI (The Pittsburgh Sleep Questionnaire Index) used for data collection. It included questions which are categorized into sleeping habits, sleep-related symptoms, and additional questions concerning bruxism. This questionnaire was randomly distributed among all college preclinical and postclinical students. Sleep bruxism diagnosis was based on self-reported data. The data were analyzed using Chi-square tests through SPSS software for Windows.* Results.* Statistical analyses revealed significant correlations between self-reported bruxism and sleeping habits including sleep initiation (*χ* ^2^ = 22.6, *p* = 0.000), continuous sleep until morning (*χ* ^2^ = 19.2, *p* = 0.001), nighttime sleep duration (*χ* ^2^ = 20.2, *p* = 0.000), and length of daytime naps (*χ* ^2^ = 28.35, *p* = 0.000). There was an association between self-reported bruxism and sleeping-related symptoms including awakening early in the morning before the usual time without a cause (*χ* ^2^ = 16.52, *p* = 0.000) and increased nightmares (*χ* ^2^ = 13.7, *p* = 0.001).* Conclusions.* Poor sleeping pattern was an important factor among dental students, who reported sleep bruxism.

## 1. Introduction

Bruxism is defined as diurnal or nocturnal parafunctional jaw muscle activity that is characterized by clenching, bracing, gnashing, and grinding of teeth [1]. Parafunctions play an important role in oral and general health [2, 3]. Muscle pain, headaches, tooth wear, temporomandibular joint disorders, and even tooth loss are some of the consequences of sleep bruxism and awake bruxism [2, 4, 5].

There are two types of bruxism: awake bruxism and a sleep bruxism with different etiologies. Awake bruxism is characterized by clenching-type activity and sleep bruxism is characterized by a combination of clenching and grinding-type activity. Most of the studies were done on sleep-related bruxism, which is more suitable for a reliable diagnosis in a scientific research setting [6].

Self-reported bruxism was recently shown to be coherently associated with stress and stress-related disorders and a possible indicator of intrapersonal or interpersonal reactivity [1, 7] or dissatisfaction in a healthy population. Furthermore, disrupted sleep was found to be associated with bruxism and orofacial pain [8], suggesting a vicious circle among these factors [9].

The association between dental practice and high levels of stress has been widely acknowledged. The origin of this stress may lie in the process of dental education [10, 11], with known stressors including time and schedule pressures, management of uncooperative patients, commercial issues, and the highly technical and intensive nature of work. These factors may result in inadequate sleep, which increases the risk of accidents and illnesses [11].

Research shows that sleep deprivation results in daytime sleepiness and impaired neurocognitive and psychomotor performances [12, 13]. There is an important relationship of sleeping patterns with learning abilities and consequent academic performance [14].

The aim of this cross-sectional study was to identify sleeping patterns among dental students and their association with self-reported bruxism in Riyadh Colleges of Dentistry and Pharmacy (RCsDP).

## 2. Methods

### 2.1. Study Design and Sample

The study protocol was approved by the Ethics Committee of Riyadh Colleges of Dentistry and Pharmacy (RCsDP) and a consent form attached to each questionnaire was signed by each student who agreed to participate in the study. A total of 549 students aged 18–24 years participated in this study (67 males and 482 females) which fulfilled the following inclusion and exclusion criteria.

*Inclusion Criteria*. All dental students with different age and gender were included aged 18–24 years.

*Exclusion Criteria*. The dental students with history of cardiac diseases and pulmonary diseases and students receiving psychiatric medication and sleep apnea are excluded from the study as these conditions may affect the sleeping pattern.

### 2.2. Data Collection

A questionnaire was distributed during lecture classes among the participants from preclinical and postclinical levels (from level 1 to level 12) of dental students by clinical instructors participating in the study for a month from November to December 2014. In the study, we use three samples: first sample from level 1 students, second sample from level 6 students, and third sample from level 12 students.

### 2.3. Questionnaire Tool

A structured questionnaire was a comprehensive instrument including items determining usual sleep/waking behaviors over the previous month. This questionnaire was adopted from a validated questionnaire of PSQI (Pittsburgh Sleep Questionnaire Index) and Arabic version of The PSQI assessing sleep quality [15, 16] and also from a questionnaire used in a previous study designed in Iran [17]. The questionnaire included additional questions asking the students if they were aware of tooth grinding during the day or night or if they were informed about tooth grinding during sleep by their partner. The students were divided into two main groups: bruxers and nonbruxers. The questions were divided into two categories. The first category questioned the students about their sleeping habits and determined the following: whether they faced any difficulty in initiating sleep (DIS), slept continuously till morning, had a fixed time for sleeping, consumed coffee before sleeping, consumed sleeping pills, or indulged in daytime naps; night sleep duration; time taken to fall asleep; and duration of daytime naps.

The second category assessed the following sleep-related symptoms: awakening early in the morning, facing difficulty in sleeping again, falling asleep in class, experiencing tiredness or sleepiness during the day, and facing difficulty awakening in the morning. In addition, the frequency of nightmares was assessed by this category.

### 2.4. Statistical Analysis

For analysis of the data, we expressed the answers as “no” (individuals who reported that they had not ground their teeth while asleep or clenched their teeth while awake in the last 30 days) and “yes” (individuals who reported that they had ground their teeth while asleep or clenched their teeth while awake in the last 30 days). Note that “sleepiness during class” was considered different from “falling asleep in class.” The former indicated a sleepy state, not actual sleep, while the latter indicated actual sleep. The subjects provided responses on a 1–5-point ordinal scale, where number 1 represented “never” and number 5 represented “almost every day or night.” Four questions required categorical answers and assessed the time of going to bed, time taken to fall asleep, duration of daytime naps, and number of nocturnal awakenings. The level of significance was set to be *p* < 0.05. All data were analyzed using chi-square tests (*χ* ^2^) through SPSS software for Windows version 17 (SPSS Inc., Chicago, IL, USA).

## 3. Results

There were 12.2% men and 87.8% women providing valid responses. From the entire study population, 168 (31.1%) students, including 25 males (37.9% of the total male population) and 143 females (30.3% of the total female population), reported awareness of tooth grinding.

### 3.1. Sleeping Habits (Table 1)

#### 3.1.1. DIS

**Figure 1:**
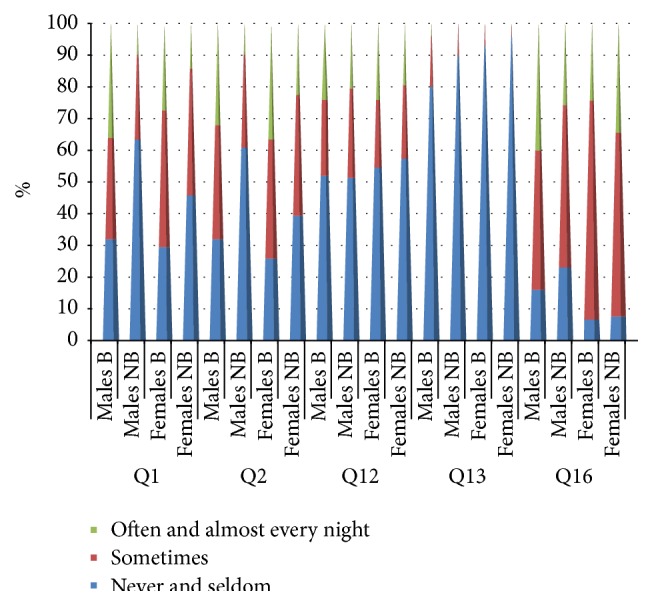
Results for questions Q1, Q2, Q12, Q13, and Q16 for male and female bruxers and nonbruxers among a cohort of dental students in Saudi Arabia. Question = Q, Q1 (Do you face any difficulty in initiating sleep?), Q2 (Do you sleep continuously until morning or is your sleep disturbed?), Q12 (Do you consume coffee in the evening?), Q13 (Do you consume sleeping pills?), and Q16 (Do you nap during the day?).

The overall proportion of students with frequent or daily DIS was significantly higher among the bruxers (28.9%, *n* = 48) than among the nonbruxers (14%, *n* = 52, chi-square test, 22.6, *p* = 0.000, *p* < 0.05). Among the men, nine bruxers (36%) complained of DIS almost every night, as opposed to only four (9.8%) nonbruxers (*χ* ^2^ = 8.56, *p* = 0.014). Among the women, 38 (27.3%) bruxers and 46 (14.2%) nonbruxers complained of DIS almost every night (*χ* ^2^ = 15.9, *p* = 0.000). There was no significant difference in the incidence of DIS between male and female bruxers (*p* < 0.05, Figure 1).

#### 3.1.2. Continuous Sleep until Morning

A significant difference was found in the number of students who always experienced disturbed sleep until morning between bruxers (36.8%, *n* = 61) and nonbruxers (20.7%, *n* = 77, *χ* ^2^ = 19.2, *p* = 0.001). With regard to gender, there was no significant difference in the number of students who slept undisturbed until morning between male bruxers and nonbruxers (*p* < 0.05), whereas a significant difference was observed between the female bruxers (25.9%, *n* = 36) and nonbruxers (39.4%, *n* = 128, *χ* ^2^ = 13.02, *p* = 0.01, Figure 1).

#### 3.1.3. Duration of Nighttime Sleep

The majority of bruxers (40.3%, *n* = 67) slept for 6–9 h, and the tendency to sleep for more than 9 h was significantly higher among bruxers (38%, *n* = 63) than among nonbruxers (20.8%, *n* = 75). Among nonbruxers, although the majority (44.2%, *n* = 159) slept for 6–9 h, the tendency to sleep for less than 6–9 h was significantly high (35%, *n* = 126, *χ* ^2^ = 20.2, *p* = 0.000).

Most male students slept for 6–9 h, with no significant difference between bruxers and nonbruxers and a tendency toward sleeping for more than 6–9 h. The majority of female students also slept for 6–9 h, although there was a significant difference between bruxers (39.6%, *n* = 55) and nonbruxers (44.2%, *n* = 140, *χ* ^2^ = 19.25, *p* = 0.001, Table 2).

#### 3.1.4. Time of Going to Bed

The time of going to bed was fixed for 28.6% (*n* = 48) bruxers and 29.9% (*n* = 110) nonbruxers, while it varied for 71.4% (*n* = 120) bruxers and 70.1% (*n* = 258) nonbruxers. There was no significant difference in the time of going to bed between bruxers and nonbruxers among both men and women, and most bruxers and nonbruxers (men and women) slept at 12 AM. Female bruxers showed a tendency to sleep early, while female nonbruxers showed a tendency to sleep late (Table 2).

#### 3.1.5. Frequency of Coffee Consumption in the Evening and Sleeping Pill Intake

The majority of students, regardless of gender or the presence or absence of bruxism, did not report coffee consumption late in the evening or sleeping pill intake, with no significant differences between groups (Figure 1).

#### 3.1.6. Time Taken to Fall Asleep Every Night

There was no significant difference in the time taken to fall asleep, regardless of gender or the presence or absence of bruxism; most students fell asleep within 30–60 min (Table 2).

#### 3.1.7. Frequency of Daytime Naps/Duration of Daytime Naps

Most students indulged in daytime naps, with some even napping twice a day, and there was no significant difference according to gender or the presence or absence of bruxism (Figure 1).

The proportion of students who napped for more than 3 h a day was significantly higher among bruxers (56.9%, *n* = 82) than among nonbruxers (37.4%, *n* = 108, *χ* ^2^ = 28.35, *p* = 0.000). Bruxers took longer naps compared with nonbruxers, regardless of gender (Table 2).

### 3.2. Sleep-Related Symptoms (Table 1)

#### 3.2.1. Awakening Early in the Morning without a Cause before the Usual Time

**Figure 2:**
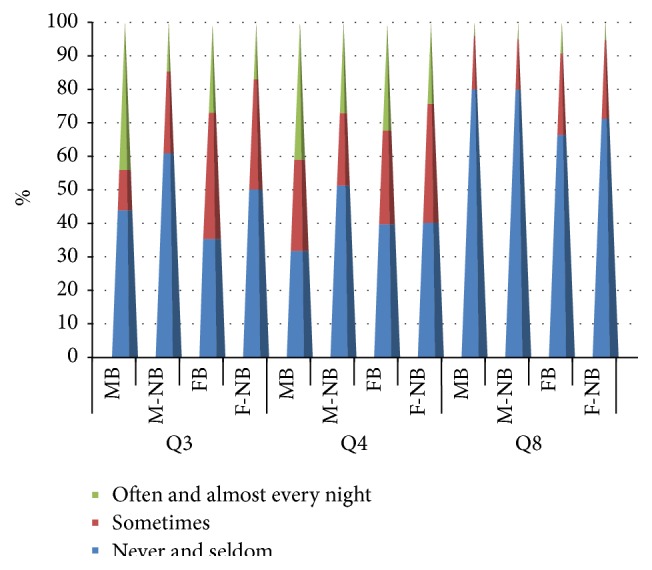
Results for questions Q3, Q4, and Q8 for male and female bruxers and nonbruxers among a cohort of dental students in Saudi Arabia. Question = Q, Q3 (Do you wake up early without a cause before the usual time?), Q4 (Do you face difficulty in falling asleep again?), and Q8 (Do you feel sleepy in class?).

Compared with nonbruxers (16.4%, *n* = 61), a significantly higher number of bruxers (30.4%, *n* = 51) woke up early in the morning without a cause (*χ* ^2^ = 16.52, *p* = 0.000). With regard to gender, 44% (*n* = 11) male bruxers frequently woke up early without a cause, while only 14% (*n* = 6) of their nonbruxer counterparts complained of the same problem (*χ* ^2^ = 7.23, *p* = 0.03, Figure 2).

Interestingly, 44% (*n* = 11) male bruxers never or seldom awoke early in the morning without a cause. Among the women, 35.5% (*n* = 50) and 50.2% (*n* = 163) bruxers and nonbruxers, respectively, never or seldom awoke early without a cause (*χ* ^2^ = 10.22, *p* = 0.006, Figure 2).

#### 3.2.2. Difficulty in Falling Asleep Again

The majority of bruxers and nonbruxers never or seldom faced difficulty in falling asleep again. The majority of male bruxers frequently or always faced difficulty in falling asleep again, while the majority of male nonbruxers never or seldom faced difficulty in falling asleep again. Most of the female bruxers and nonbruxers seldom or never faced difficulty in falling asleep again (*χ* ^2^ = 4.22, *p* = 0.12, Figure 2).

#### 3.2.3. Feeling Sleepy in Class

The majority of students never or seldom felt sleepy in class, regardless of gender or the presence of absence of bruxism. Although the difference was not significant, a higher proportion of female bruxers felt sleepy in class compared with their nonbruxer counterparts (*χ* ^2^ = 2.16, *p* = 0.34, Figure 2).

#### 3.2.4. Feeling Tired and Sleepy during the Day

**Figure 3:**
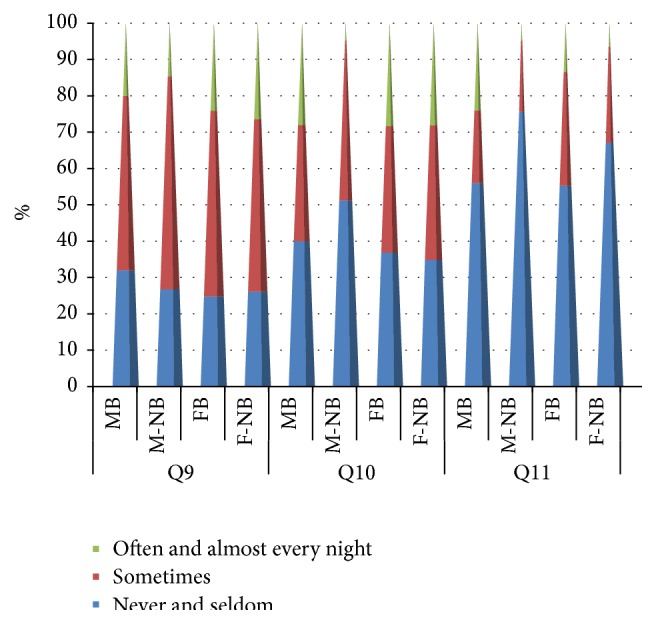
Results for questions Q9, Q10, and Q11 for male and female bruxers and nonbruxers among a cohort of dental students in Saudi Arabia. Question = Q, Q9 (Do you feel tired and sleepy during the day?), Q10 (Do you face difficulty in awakening in the morning?), and Q11 (Do you experience nightmares?).

There were no significant differences in the number of bruxers and nonbruxers who felt tired and sleepy during the day (*χ* ^2^ = 0.24, *p* = 0.89, Figure 3).

#### 3.2.5. Difficulty in Awakening in the Morning

A similar proportion of bruxers and nonbruxers reported difficulty in awakening in the morning. Among the men, a significantly higher (28%, *n* = 7) proportion of bruxers reported difficulty in awakening in the morning compared with that of nonbruxers (4.9%, *n* = 2, *χ* ^2^ = 7.06, *p* = 0.029). No significant difference was observed between female bruxers and nonbruxers (*χ* ^2^ = 0.875, *p* = 0.646, Figure 3).

#### 3.2.6. Incidence of Nightmares

The incidence of frequent or almost daily nightmares was significantly higher for bruxers (14.9%, *n* = 25) than for nonbruxers (6.2%, *n* = 23, *χ* ^2^ = 13.7, *p* = 0.001). More precisely, compared with that of male nonbruxers (4.9%, *n* = 2), a higher proportion of male bruxers (24%, *n* = 6) experienced nightmares often or almost daily, with no significant difference (*χ* ^2^ = 5.56, *p* = 0.06). On the other hand, a lower proportion of female bruxers (13.5%, *n* = 19) experienced nightmares often or almost daily compared with that of female nonbruxers (6.5%, *n* = 21), with no significant difference (*χ* ^2^ = 8.56, *p* = 0.014, Figure 3).

#### 3.2.7. Falling Asleep in Class

In total, 20% (*n* = 33) bruxers and 14.6% (*n* = 54) nonbruxers fell asleep in class, with no significant difference. Among the men, 80% (*n* = 20) bruxers and 87.2% (*n* = 34) nonbruxers did not sleep in class; the corresponding values were 79.7% (*n* = 110) and 84.9% (*n* = 276), respectively, for the women (*χ* ^2^ = 2.41, *p* = 0.12).

## 4. Discussion

The present study demonstrated the disturbance of sleeping habits and sleeping related symptoms among the students who reported bruxism in RCsDP.

A significant association was found in bruxism, with 28.9% bruxers experiencing regular DIS compared with 14% nonbruxers. These values were higher than those in a study conducted in Finland among media personnel with irregular working hours; only 11.9% bruxers experienced regular DIS [8]. Dental students experience higher stress levels than do medical students [18–20]. Insomnia or DIS is related to stressful events when mediated by certain predisposing emotional factors such as a personality trait of neuroticism, internalization of stressful events [21, 22], and inadequate coping mechanisms [23, 24].

There is a positive association of psychosocial factors such as reactions to frustrating experiences, anxiety, and stress with self-reported bruxism [25]. Neither stress nor personality was associated with the intensity of bruxism measured by electromyography, and those nonspecific personality characteristics of individuals with bruxism have never been identified [26].

Moreover, according to Schneider et al. (2007), less positive coping strategies were exhibited by individuals with sleep bruxism than by those without [27].

A significant correlation was observed between disturbed sleep until morning and bruxism, with 36.8% bruxers complaining of regularly disturbed sleep. This finding is consistent with that of a previous study where individuals with sleep bruxism reported a significantly lower frequency of deep sleep compared with the normal subjects [28].

The present study found that, for bruxism and nighttime sleep duration, bruxers reported significantly longer sleep durations compared with nonbruxers. Individuals who slept for longer durations exhibited higher levels of subjective sleepiness after sleep deprivation, while those who slept for shorter durations exhibited no significant increase in sleepiness levels [29]. With regard to the duration of daytime naps, 56.9% bruxers napped for longer than 3 h, as opposed to only 37.4% nonbruxers.

The World Health Organization recommended a minimum of eight hours' sleep per night as ideal amount of a good quality on night sleep. Moreover, individuals who sleep for shorter durations tend to develop subclinical hypomania [30], whereas those who sleep longer tend to be depressed more frequently or almost daily [31–33]. Bruxers were found to be more anxious and depressed compared with nonbruxers [34].

When asking about the frequency of nightmares, there were 14.9% bruxers reporting nightmares often or almost every night. This value was higher than that in a previous study where 8.5% subjects experienced bruxism at least one night every week, which is within the range of 6%–20% reported for the general population [35]. Nightmares and bruxism can be triggered by occasional stress [36]. Bruxism was also suggested to be related to anxiety sensitivity, regardless of the severity of anxiety or depressive symptoms. The present study found an association between self-reported bruxism, sleep disturbance, and habitual sleep efficiency.

The physiology and pathology of self-reported bruxism is not widely known, but emotional problems, anxiety, and stress can be risk factors for this parafunction. The student life is full of pressures which may lead to stress and also lead to the release of tensions by clenching the teeth [37].

In this study a structural questionnaire was used which was based on a validated questionnaire of The PSQI (The Pittsburgh Sleep Questionnaire Index). The PSQI is an instrument which was validated in different languages and widely used as a simple tool in different studies concerned with sleep [38]. Similarly it is used as a tool in the study of Dutch adolescents [39]. Another limitation of this study was that there was an imbalance between males and females participating in the study as the female volunteers in our study were more than males.

The other limitation was the use of the questionnaire as a tool in this study for evaluation of sleep bruxism and awake bruxism from answering the questions as self-reported or partner-reported. Self-reported sleep bruxism and awake bruxism results were previously used in another study [4].

Evaluations of sleep and its related symptoms by polysomnography are more accurate but on the other hand involve more equipment, resulting in increased financial costs [40, 41]. Evaluation using electronic instruments is more difficult to be applied on a large number of participants and the use of The PSQI is commonly recommended for epidemiological studies [42, 43].

Cross-sectional studies are commonly used in epidemiological studies to know risk factors and associations, but not to evaluate causes. Longitudinal studies on this subject, with representative samples, should be performed [42].

## 5. Conclusions

The findings of the present study reveal that poor sleeping pattern is an important factor among dental students, who reported sleep bruxism; therefore, the management of bruxism may modify or improve sleeping patterns and we suggest further researches for studying this condition.

## Figures and Tables

**Table 1:** Sleeping habits and sleeping disturbances in bruxers and nonbruxers among dental students in Saudi Arabia.

Sleeping habits & sleeping disturbances		Bruxer	Nonbruxer	*χ* ^2^	*p* value
Q1: Do you face any difficulty in initiating sleep?	Never/Seldom	29.5% (49)	47.7% (177)	22.6	0.000^*∗*^
	Sometimes	41.6% (69)	38.2% (142)		
	Almost every night	28.9% (48)	14% (52)		
	Total	100% (166)	100% (371)		

Q2: Do you sleep continuously until morning or is your sleep disturbed?	Always disturbed	36.8% (61)	20.7% (77)	19.2	0.001^*∗*^
	Sometimes disturbed	36.7% (61)	37.2% (138)		
	Continuous sleep till morning	26.5% (44)	42.1% (156)		
	Total	100% (166)	100% (371)		

Q3: Do you wake up early without a cause before the usual time?	Never/Seldom	36.3% (61)	51.5% (190)	16.3	0.000^*∗*^
	Sometimes	33.3% (56)	32.1% (119)		
	Almost every day/night	30.4% (51)	16.4% (61)		
	Total	100% (168)	100% (370)		

Q4: Do you face difficulty in falling asleep again?	Never/Seldom	38.2% (60)	42.3% (139)	4.22	0.12
	Sometimes	28.7% (45)	33.4% (110)		
	Almost every day/night	33.1% (52)	24.3% (80)		
	Total	100% (157)	100% (329)		

Q5: How many hours do you sleep every night?	3–6 h	21.7% (36)	35% (126)	20.2	0.000^*∗*^
	6–9 h	40.3% (67)	44.2% (159)		
	More than 9 h	38% (63)	20.8% (75)		
	Total	100% (166)	100% (360)		

Q6: Do you have a fixed time of going to bed every night?	Yes	28.6% (48)	29.9% (110)	0.09	0.76
	No	71.4% (120)	70.1% (258)		
	Total	100% (168)	100% (368)		

Q7: Approximately when do you go to bed every night?	Before 12 o'clock	36.2% (22)	34.1% (44)	12.1	0.44
	At 12 o'clock	55.8% (34)	44.3% (57)		
	After 12 o'clock	8% (5)	21.6% (28)		
	Total				

Q8: Do you feel sleepy in class?	Never/Seldom	68.9% (115)	72.6% (267)	2.16	0.34
	Sometimes	22.8% (38)	22.3% (82)		
	Almost every day/night	8.3% (14)	5.1% (19)		
	Never/Seldom	68.9% (115)	72.6% (267)		

Q9: Do you feel tired and sleepy during the day?	Never/Seldom	26.2% (44)	26.1% (97)	0.24	0.89
	Sometimes	50.6% (85)	48.8% (181)		
	Almost every day/night	23.2% (39)	25.1% (93)		
	Total	100% (168)	100% (371)		

Q10: Do you face difficulty in awakening in the morning?	Never/Seldom	37.5% (63)	37% (138)	0.57	0.75
	Sometimes	34.5% (58)	37.5% (140)		
	Almost every day/night	28% (47)	25.5% (95)		
	Total	100% (168)	100% (373)		

Q11: Do you experience nightmares?	Never/Seldom	55.3% (93)	68.5% (254)	13.72	0.001^*∗*^
	Sometimes	29.8% (50)	25.3% (94)		
	Almost every day/night	14.9% (25)	6.2% (23)		
	Total	100% (168)	100% (371)		

Q12: Do you consume coffee in the evening?	Never/Seldom	54.2% (91)	56.8% (209)	1.9	0.39
	Sometimes	21.4% (36)	23.9% (88)		
	Almost every night	24.4% (41)	19.3% (71)		
	Total	100% (168)	100% (368)		

Q13: Do you consume sleeping pills?	Never/Seldom	91.1% (153)	95.4% (353)	3.9	0.14
	Sometimes	7.7% (13)	4.1% (15)		
	Almost every night	1.2% (2)	0.5% (2)		
	Total	100% (168)	100% (370)		

Q14: Do you fall asleep in class?	Yes	20% (33)	14.6% (54)	2.41	0.12
	No	80% (132)	85.4% (315)		
	Total	100% (165)	100% (369)		

Q15: How long do you take to fall sleep [*sic*] every night?	30 min or less	48.3% (71)	59.8% (186)	12.9	0.17
	60 min or more	51.7% (76)	40.2% (125)
	Total	100% (147)	100% (311)

Q16: Do you nap during the day?	Never	21.0% (35)	21.8% (80)	2.1	0.35
	Seldom	43.1% (72)	36.8% (135)		
	Sometimes	35.9% (60)	41.4% (152)		
	Total	100% (167)	100% (367)		

Q17: How many naps do you take during the day?	No naps	7.1% (13)	9.3% (34)	3.9	0.70
	One nap	64.2% (106)	56.6% (206)		
	More than one nap	28.7% (44)	34.1% (124)		
	Total	100% (163)	100% (364)		

Q18: What is the duration of your daytime naps?	1-2 h or less	34.8% (50)	37.7% (109)	28.35	0.000^*∗*^
	2-3 h	8.3% (12)	24.9% (72)		
	More than 3 h	56.9% (82)	37.4% (108)		
	Total	100% (144)	100% (289)		

^*∗*^ *p* < 0.05.

**Table 2:** Sleeping habits in male and female bruxers and nonbruxers among dental students in Saudi Arabia.

Sleeping habits		MB	M-NB	*χ* ^2^	*p* value	FB	F-NB	*χ* ^2^	*p* value
How many hours do you sleep every night?	3–6 h	8% (2)	13.2% (5)	0.79	0.85	24.5% (34)	37.8% (120)	19.3	0.001^*∗*^
	6–9 h	48% (12)	42.1% (16)			39.5% (55)	44.2% (140)		
	More than 9 h	44% (11)	44.7% (17)			36% (50)	18% (57)		
	Total	100% (25)	100% (38)			100% (139)	100% (317)		

Do you have a fixed time of going to bed every night?	Yes	20% (5)	22% (9)	0.04	0.55	30.5% (43)	30.3% (98)	0.001	0.24
	No	80% (20)	78% (32)			69.5% (98)	69.7% (225)		
	Total	100% (25)	100% (41)			100% (141)	100% (323)		

Approximately when do you go to bed every night?	Before 12 o'clock	0% (0)	10% (1)	4.74	0.32	43.1% (22)	36.6% (42)	9.39	0.67
	At 12 o'clock	100% (9)	60% (6)			47.1% (24)	41.7% (48)		
	After 12 o'clock	0% (0)	30% (3)			9.8% (5)	21.7% (25)		
	Total	100% (9)	100% (10)			100% (51)	100% (115)		

How long do you take to fall asleep every night?	30 min or less	47.6% (10)	74.1% (20)	6.81	0.45	48% (60)	58.6% (164)	12.36	0.19
	60 min or more	52.4% (11)	25.9% (7)			52% (65)	41.4% (116)		
	Total	100% (21)	100% (27)			100% (125)	100% (280)		

How many naps do you take during the day?	No naps	16% (4)	21.9% (9)	2.82	0.59	6.5% (9)	7.6% (24)	5.54	0.24
	One nap	44% (11)	48.8% (20)			68.1% (94)	57.4 (182)		
	More than one nap	40% (10)	29.3% (12)			25.4% (35)	35% (111)		
	Total	100% (25)	100% (21)			100% (138)	100% (317)		

What is the duration of your daytime naps?	1-2 h or less	19% (4)	46.4% (13)	10.39	0.03	37.2% (45)	36.7% (94)	27.3	0.000^*∗*^
	2-3 h	19% (4)	10.7% (3)			5.8% (7)	26.2% (67)		
	Total	100% (21)	100% (28)			100% (121)	100% (256)		

^*∗*^ *p* < 0.05.
